# Measuring the Science of Caring: A Patient‐Centred Predictive Model for the Caring Interaction

**DOI:** 10.1111/scs.70110

**Published:** 2025-09-09

**Authors:** Regina Allande‐Cussó, María Alejandra Pinero‐De Plaza, Juan Gómez‐Salgado, Ana María Porcel‐Gálvez

**Affiliations:** ^1^ Department of Nursing University of Seville Seville Spain; ^2^ Caring Future Institute, College of Nursing and Health Sciences Flinders University, Adelaide Bedford Park South Australia Australia; ^3^ Department of Sociology, Social Work and Public Health University of Huelva Huelva Spain

**Keywords:** caring interaction, NIC_CA‐patient, nurse–patient relationship, nursing science, patient‐centred care, predictive model

## Abstract

**Background:**

The nurse–patient relationship is central to quality nursing care, yet its impact remains difficult to quantify. While existing models assess caring competencies from the perspective of nursing students and professionals, there is a lack of validated instruments incorporating direct patient feedback.

**Objective:**

This study aimed to develop and validate the Nursing Interaction in Caring_Competence Assessment—Patient (NIC_CA‐Patient) tool, a patient‐centred instrument designed to measure caring interaction in nursing practice and establish a predictive model of its development from the patient's perspective.

**Methods:**

A cross‐sectional study was conducted in eight hospitals within the Andalusian Health Care System, involving 1060 patients admitted to internal medicine units. The NIC_CA‐Patient tool was adapted from the Caring Nurse–Patient Interactions (CNPI) scale and validated through exploratory factor analysis (EFA) and partial least squares structural equation modelling (PLS‐SEM).

**Results:**

The final model identified three key dimensions—therapeutic relationship, problem management and adaptation—explaining 79% of the variance in patient‐reported experiences. The predictive model demonstrated strong reliability (Cronbach's α = 0.97) and excellent model fit indices (SRMR = 0.03, AVE > 0.7).

**Conclusion:**

The NIC_CA‐Patient tool provides a quantifiable and predictive measure of caring interaction from the patient's perspective. Its implementation in clinical practice and research can enhance patient‐centred care by identifying relational factors that improve health outcomes and patient experience.

## 1 Introduction

The nurse–patient relationship is a fundamental aspect of nursing practice, forming the basis of high‐quality care and patient‐centred interactions. This dynamic relationship, characterised by trust, empathy, respect and communication, significantly influences patient outcomes, emotional well‐being and overall healthcare experiences [1, 2, 3, 4]. Despite the increasing recognition of its importance, quantitative evidence linking the nurse–patient relationship to measurable patient outcomes remains limited, creating a methodological gap that this study aims to address [5].

In contemporary nursing theories, the Person‐Centred Care Framework [6] and the Fundamentals of Care Framework [7] underscore the nurse–patient relationship as central to patient care. These frameworks advocate for respecting patient preferences, addressing physical and emotional needs and fostering an interdisciplinary approach. However, these models largely focus on healthcare professionals' perspectives, leaving a critical gap in understanding how patients themselves perceive and experience these interactions [8, 9, 10].

Recent conceptual advancements have redefined the nurse–patient relationship as a ‘caring interaction’, moving beyond task‐based care to emphasise a reciprocal and relational process. This approach recognises that patients are not passive recipients of care but active participants whose engagement influences care outcomes [11, 12]. The Nursing Interventions Classification (NIC 5000) formalises caring interaction as a core nursing intervention, highlighting communication, ethical respect and emotional support as integral components [13].

The impact of caring interactions on patient‐reported outcomes (PROMs) and experiences (PREMs) is increasingly evident [8, 9, 10]. Effective nurse–patient interactions are associated with higher patient satisfaction, better adherence to treatment and lower anxiety and stress [14, 15, 16, 17]. An effective care interaction can significantly improve PROMs, resulting in increased patient satisfaction, improved treatment adherence and reduced anxiety and stress during the care process [17]. In addition, when the nurse–patient relationship is based on trust and mutual respect, it facilitates more open and honest communication, allowing patients to express their concerns, preferences and expectations more clearly [18]. This, in turn, contributes to more personalised care focused on the patient's needs, which improves health outcomes and the overall experience of care [19]. However, there is still a lack of quantitative evidence on measurable outcomes of the impact of the nurse–patient relationship on health outcomes, such as level of anxiety, pain or satisfaction with the care received [5].

Several instruments have been developed to measure nurse–patient interaction competence or specific attributes such as empathy, communication or professional presence [20]. Notable among these are the Caring Behaviours Inventory (CBI) [21], the Caring Dimensions Inventory (CDI) [22], and the Caring Nurse–Patient Interactions (CNPI) Scale [23]. These tools have contributed significantly to understanding caring in nursing; however, they tend to focus on either specific behavioural aspects or use self‐report data from professionals. Moreover, many of them offer a cross‐sectional snapshot rather than a developmental or process‐oriented view of caring.

A growing body of literature calls for renewed attention to patients' voices as key informants in assessing the quality of caring interactions [23]. While some existing tools include patient‐reported items, few are explicitly designed to capture the developmental sequence of the caring interaction as experienced by the patient. Furthermore, there is limited integration between such instruments and foundational theoretical frameworks such as Person‐Centred Care [24] or the Fundamentals of Care Framework [7], which could enhance their pedagogical and clinical utility. Among these instruments, the Caring Nurse–Patient Interactions (CNPI) questionnaire stands out due to its rigorous process of construction and cultural adaptation, its optimal psychometric properties (with Cronbach's *α* = 0.97; correlations between subscales of 0.53 and 0.89; and Pearson coefficients ranging between −0.02 and 0.32) [22, 25]. The cross‐cultural adjustment and initial psychometric validation of the CNPI scale in Spain resulted in a 28‐item scale, with five factors explaining 60.48% of the variance, and a Cronbach's *α* value of 0.9 [2, 26]. Based on this adjusted tool, the *Nursing Interaction in Caring_Competence Assessment* (NIC_CA) tool was designed to validate a predictive model of the development of the caring interaction from the perspective of the nursing students [2]. The model establishes a linear and sequential relationship between five composites, which would be assimilated to the phases of the caring interaction, of ‘needs’, therapeutic relationship’, ‘problem solving’, ‘self‐confidence’ and ‘mental balance’, measured by the items of the scale. In the same way, another predictive model of the development of the caring interaction, based on the development of the *Nursing Interaction in Caring_Competence Assessment—Professionals* (NIC_CA‐Prof) tool, was designed from the perspective of Registered Nurses [27]. This new model also establishes a linear and sequential relationship between only four composites of ‘basic nursing care’, ‘therapeutic relationship’, ‘problem management’ and ‘adaptation’. No other studies have reported a predictive model of the same construct from the patient's perspective. These studies have focused on assessing interaction competence from nursing students' and registered nurses' perspectives, yet a predictive model from the patient's perspective is missing [2, 27]. This gap limits the ability to systematically improve nurse–patient interactions based on patient experiences and their effect on practice as supported by the literature [28, 29, 30].

This study seeks to fill this methodological gap by developing and validating a predictive model of caring interactions from the patient's perspective. Grounded in the Fundamentals of Care and Person‐Centred Care frameworks, this model aims to capture patients' perceptions of essential caring behaviours, therapeutic alliances, problem‐solving interactions and adaptation to health conditions. Understanding these patient‐driven predictors of effective care interactions will allow healthcare professionals to refine their practice and tailor interventions that enhance both clinical outcomes and patient experiences. Thus, the primary objective of this study is to conduct an initial psychometric exploration of the *Nursing Interaction in Caring_Competence Assessment—Patient* (NIC_CA‐Patient) instrument through exploratory factor analysis (EFA), and to develop a patient‐centred predictive model of the caring interaction based on the factors identified. The proposed model is based on four hypotheses, structured in a sequential framework:

- The caring interaction begins with addressing fundamental patient‐centred care needs.
- A therapeutic alliance is essential in supporting the patient's recovery process.
- Caring interactions strengthen as nurses assist patients in managing health challenges.
- Adaptation occurs when patients demonstrate an ability to integrate care into their health trajectory.

## 2 Methods

### 2.1 Design

A descriptive cross‐sectional study was conducted in two distinct phases. The initial phase involved the preliminary psychometric study of the NIC_CA‐Patient tool, followed by a second phase that aimed to develop a predictive model. A descriptive cross‐sectional design was selected as it allows for robust psychometric evaluation and predictive modelling in healthcare research [31]. Following established guidelines for the validation of patient‐reported measures [32], this study aimed to establish a reliable tool for assessing caring interactions from the patient's perspective.

### 2.2 Population and Sample

The study was conducted within the Andalusian Health Care System in southern Spain in 2024, using a consecutive sampling procedure. It focused on patients admitted to internal medicine units across eight hospitals: Hospital Universitario Virgen del Rocío, Hospital Virgen Macarena, Hospital Universitario Virgen de Valme, Hospital Universitario San Juan de Dios–Bormujos, Hospital Universitario Reina Sofía, Hospital Universitario Puerta del Mar, Hospital San Carlos and Hospital Universitario Virgen de las Nieves.

Eligible participants were adults (≥ 18 years old) admitted to internal medicine wards who were capable of providing informed consent. Patients with severe health conditions that limited their ability to participate reliably and those who declined to participate were excluded.

For psychometric validation, 5–10 subjects per item are required [33]. Based on this criterion, a minimum of 140 patients was estimated for the 28‐item instrument. The final sample size of 1060 participants significantly exceeded the 1000‐subject threshold, which is considered excellent for factor analysis [34].

### 2.3 Variables and Instruments

The study included demographic variables (gender and age), clinical data (reason for admission) and institutional variables (hospital), as well as the NIC_CA‐Patient questionnaire variables (individual item scores and total scale score).

### 2.4 Development and Adaptation of the NIC_CA‐Patient Instrument

The NIC_CA‐Patient questionnaire was developed from a previously validated Spanish version of the Caring Nurse–Patient Interactions (CNPI) scale by Cossette et al. [22, 25], which was culturally adapted and validated in Spain with five factors explaining 60.48% of the variance and a Cronbach's α of 0.9 [2, 26]. Scoring followed a Likert scale (1 = not at all competent to 5 = extremely competent). The total score was calculated as the mean score across all items.

The cross‐cultural adaptation process followed international guidelines and included forward translation by two bilingual experts, backward translation by independent translators and a final consensus review by a multidisciplinary panel in a previous study [35]. Written permission for use and adaptation of the original scale was obtained from the authors. The Spanish version initially consisted of 70 items, distributed across 10 dimensions. In a subsequent psychometric study involving undergraduate nursing students [2], EFA was used to reduce the instrument to 28 items Supporting Information S1. These items were reorganised into five composites in the predictive model [2] and served as the basis for developing the NIC_CA‐Patient version in this study.

In this sense, each of these 28 items was carefully reformulated to ensure linguistic clarity, conceptual relevance, and accessibility for hospitalised adult patients. A content validity assessment was conducted through a panel of five experts in nursing care, all holding at least a Master's‐level academic qualification. Three of the five experts were women, with a mean age of 45 years (SD = 2.3). Experts were selected by convenience sampling from among the management and coordination teams of the Internal Medicine units in the participating hospitals. A structured review session was held in December 2024, during which each item was discussed and potential improvements were identified. The final wording of the 28 items was established by majority consensus. The final list of adapted items used in the present study, including minor linguistic modifications, is available in Supporting Information S1.

### 2.5 Data Collection

Data collection was conducted over 6 months (January–June 2024) using LimeSurvey software, ensuring secure and confidential data handling.

Recruitment and interviewing involved 18 registered nurses from the HUMANCUIDA research project (Instituto de Salud Carlos III, Spanish Ministry of Health, PI 22/00373) and 16 trained interviewers from a specialised healthcare recruitment company. Structured interview protocols were implemented to minimise interviewer bias. Interviewers participated in two 5‐h training sessions, including simulated interviews for standardisation and reliability assessment. To ensure data consistency, periodic monitoring and inter‐rater reliability checks were conducted [36].

Interviews were conducted face‐to‐face, using tablets for real‐time data entry. All participants provided informed consent before participation. Only stable patients were interviewed to ensure ethical integrity.

### 2.6 Data Analysis

A univariate descriptive analysis of the data was conducted using SPSS v29 [37]. Percentages, means, and standard deviations were computed for each variable to describe the sample. Missing data were handled using listwise deletion, as the proportion of missing responses was minimal (< 5%) [38]. The Kolmogorov–Smirnov test indicated that the data did not follow a normal distribution (*p* < 0.05).

EFA was conducted after calculating the Kaiser–Meyer–Olkin statistic and Bartlett's test of sphericity, using Principal Axis Factoring, as this method is robust to moderate violations of normality. A varimax rotation was applied, and items with loadings below 0.50 were excluded from the final factor solution [39]. The number of factors to retain was determined using Kaiser's criterion (eigenvalues > 1) and visual inspection of the scree plot [40]. Reliability was assessed using both Cronbach's Alpha and McDonald's Omega, with the latter being preferred when tau equivalence was not met, since it reduces the risk of underestimating reliability [41].

A Partial Least Squares Structural Equation Modelling (PLSc‐SEM) regression analysis was carried out using ADANCO v2.4 software [42] to examine predictive relationships within the factors identified in the EFA analysis. The PLSc‐SEM algorithm was applied to adjust reflective construct correlations for consistency with factor models [43]. Internal consistency was evaluated using Cronbach's alpha, Dijkstra–Henseler's Rho A and composite reliability (with cut‐off values > 0.7) and convergent validity was confirmed with an Average Variance Extracted (AVE) greater than 0.5. Variance Inflation Factor (VIF) (with cut‐off values < 3.3), beta coefficients and other indices like Coefficient of Determination (R^2^), Standardised Root Mean Square Residual (SRMR) (with cut‐off values < 0.08) and Heterotrait‐Monotrait Ratio (HTMT) (with cut‐off values ≤ 0.85) were used to assess multicollinearity, effect sizes and model fit following established thresholds [44].

### 2.7 Ethical Considerations

Ethical approval was obtained from the Ethics Committee of the Andalusian government (approval code: 0840‐N‐22).

All participants provided voluntary, informed consent. Confidentiality and data protection followed the Spanish Organic Law 3/2018 and the General Data Protection Regulation ([45]). The study adhered to the ethical guidelines outlined in the Declaration of Helsinki [46]. Permission was obtained from the original author to use the CNPI questionnaire as the foundation for the NIC_CA‐Patient tool.

## 3 Results

### 3.1 Descriptive Analysis

The questionnaire was tested in a sample of 1060 patients admitted to internal medicine wards. The results included demographic characteristics (mean age of 71.5 years, SD = 16.4; 48% were women, corresponding to 509 individuals), institutional information (hospital of admission) and clinical data (reason for admission). Regarding the hospital of admission, 19.9% of the participants were recruited from Hospital Virgen Macarena (*n* = 211), 18.9% from Hospital Universitario Virgen del Rocío (*n* = 200), 18.1% from Hospital Universitario Reina Sofía (*n* = 192), 13.3% from Hospital Universitario Virgen de Valme (*n* = 141), 8.0% from Hospital Universitario Puerta del Mar (*n* = 85), 7.9% from Hospital Universitario San Juan de Dios–Bormujos (*n* = 84), 7.4% from Hospital San Carlos (*n* = 78) and 6.5% from Hospital Universitario Virgen de las Nieves (*n* = 69). Concerning the reasons for admission, respiratory conditions were the most prevalent, accounting for 52.2% of the sample (*n* = 553). These were followed by cardiological conditions with 28.1% (*n* = 298), and processes related to fever and infections with 15.9% (*n* = 169). Other reasons, such as abdominal‐digestive, oncological, traumatological or neurological conditions, were grouped under ‘other causes’ and represented only 3.8% of the sample (*n* = 40).

### 3.2 Psychometric Study

The Kaiser–Meier–Olkin test obtained a value of 0.97, and the Bartlett test of sphericity obtained a statistically significant value (*p* < 0.001). Using EFA, a dimensional matrix of 21 items and 3 factors explaining 79% of the variance was extracted. The three factors were named by the research team: therapeutic relationship, problem management and adaptation (Table 1).

**TABLE 1:** Dimensional structure and factor loadings identified in the EFA.

CNPI Scale*	Therapeutic relationship	Problem management	Adaptation
CNPI 7	,680**	,245	,234
CNPI 9	,275	,687**	,372
CNPI 10	,281	,725**	,235
CNPI 11	,302	,705**	,247
CNPI 12	,295	,739**	,231
CNPI 18	,349	,696**	,258
CNPI 33	,224	,648**	,283
CNPI 34	,231	,593**	,168
CNPI 35	,258	,530**	,287
CNPI 36	,383	,692**	,321
CNPI 45	,665**	,530	,317
CNPI 51	,654**	,507	,366
CNPI 52	,616**	,550	,377
CNPI 55	,585**	,166	,299
CNPI 64	,555**	,418	,547
CNPI 65	,521**	,346	,322
CNPI 66	,341	,255	,612**
CNPI 67	,271	,166	,521**
CNPI 68	,398	,368	,676**
CNPI 69	,395	,224	,735**
CNPI 70	,231	,259	,704**

* Original items from the Spanish version of the CNPI Questionnaire [26].

** Bolded values indicate the highest loading per item.

From a total of 28 items, 7 were eliminated because they had weights with a value lower than 0.4 or were redundant because they shared more variance between them than directly explained by a common factor [39]. The reliability study yielded a Cronbach's α value = 0.97 for the total tool, and a McDonald's Omega value = 0.92.

### 3.3 Predictive Model Design

**FIGURE 1:**
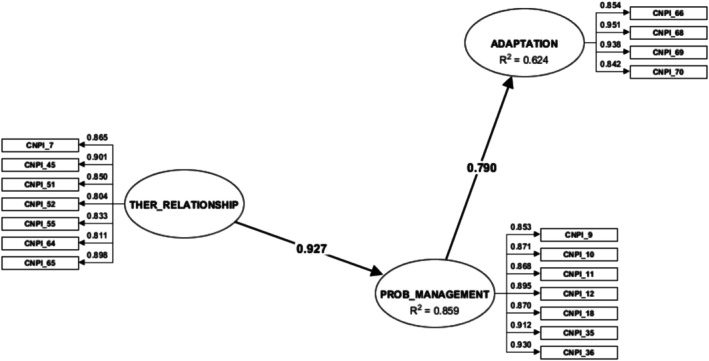
Predictive model of caring interaction from the patient's perspective, illustrating the three latent composites (therapeutic relationship, problem management and adaptation) with their observed items. Standardised factor loadings are shown on the arrows linking items to each composite, while the main structural pathways are indicated with β coefficients. The value displayed within each composite corresponds to its R^2^.

After identifying the theoretical relationships between the three factors, based on the *Fundamentals of Care* Framework [7] and *Person‐Centred Care* Framework [6], a partial least squares regression analysis (PLSc‐SEM) was run to test the hypotheses of the predictive model (Figure 1). To obtain the predictive model of caring interaction, a path diagram was designed that related the three composites (previously called factors), keeping their names. In the design of the relationships, the hypotheses described for the present study were considered.

The 21 items extracted by EFA (Table 1) were included in the initial parameter calculation with PLSc, although items CNPI 33, CNPI 34, and CNPI 67 were subsequently removed from the model due to collinearity (VIF values > 3.3) or weights below 0.4. Figure 1 describes the final version of the NIC_CA‐Patient Tool Supporting Information S2 and the predictive model of the caring interaction from the patient's perspective:

The values of the adjustment parameters provided by the PLS‐SEM method were generally good (*p* < 0.05): SRMR = 0.03 (critical value < 0.08); HTMT ≤ 0.85 (critical value ≤ 0.85); AVE ≥ 0.7 (critical value ≥ 0.5). The reliability analysis yielded values > 0.8 for Cronbach's *α* and composite reliability (critical value > 0.7). In the cases of each construct, the *R* ^2^ value obtained for each of them was > 0.6, indicating considerable levels of predictive accuracy (see Figure 1, with values inserted in each construct). All the loadings of each item within its construct were ≥ 0.8 (see Figure 1). As shown in Figure 1, each composite is represented by its observed items, with the corresponding standardised factor loadings indicated on the arrows. The structural pathways between composites are depicted with directional arrows, annotated with their β coefficients, indicating the strength of predictive relationships. Within each composite, the R^2^ value is reported, reflecting the proportion of variance explained by the model. The beta coefficients were 0.92 and 0.79, indicating a strong association in the occurrence of each pair of constructs (*p* < 0.01). Therefore, from the patient's perspective, when the composite therapeutic relationship occurs, the composite problem management occurs 0.92 times more; similarly, when the composite problem management occurs, the composite adaptation occurs 0.79 times more.

## 4 Discussion

This study presents the first validated predictive model of caring interactions grounded in patient‐reported experience. Unlike previous tools developed from the perspective of students or professionals, the NIC_CA‐Patient captures the sequential development of caring relationships as experienced by patients. The model identifies a three‐phase progression—therapeutic relationship, problem management and adaptation—offering novel insights into how patients perceive and integrate care interactions throughout their health trajectory. This patient‐centred framework extends existing theories and provides practical guidance for tailoring care strategies that enhance relational quality and patient adaptation.

The NIC_CA‐Patient model highlights the sequential nature of care interactions, where a strong therapeutic relationship predicts better problem management, and effective problem management predicts successful adaptation. This sequential process underscores the progressive nature of patient adaptation in healthcare settings and aligns with contemporary person‐centred care frameworks that emphasise relational aspects of care as integral to patient outcomes [6, 7].

### 4.1 Theoretical Positioning and Contribution of the Predictive Model

The proposed predictive model, comprising the composites of *Therapeutic Relationship*, *Problem Management*, and *Adaptation*, aligns with key tenets of existing nursing theories, particularly the *Fundamentals of Care Framework* and the *Person‐Centred Nursing Framework*. These frameworks emphasise relational care, shared decision‐making, and the dynamic interaction between physical, psychosocial and relational care needs [7, 47]. The composite *therapeutic relationship* captures the essence of emotional presence and empathy, foundational to both frameworks. *problem management* reflects the collaborative approach to identifying and addressing patient concerns, resonating with the fundamentals of care domain of ‘integration of care’. Finally, *adaptation* encompasses the patient's capacity to regain balance and autonomy in health, a key outcome in person‐centred models.

This model is also strongly anchored in the *Person‐Centred Nursing Framework*, as proposed by McCormack and McCance [47], which conceptualises care through four interrelated domains: *prerequisites*, *care environment*, *person‐centred processes* and *outcomes*. In this context, the *therapeutic relationship* composite reflects the *person‐centred processes* domain, as it involves authentic engagement, sympathetic presence and working with patients' beliefs and values. *Problem management* corresponds to the *care environment* domain, where supportive systems and effective care cultures enable nurses to respond to patients' evolving needs. *Adaptation*, in turn, is consistent with the *outcomes* domain, where successful caring interactions promote greater patient involvement, satisfaction and well‐being.

Prior predictive models assessing caring interactions have primarily focused on nursing students and professionals rather than patients. The NIC_CA tool for nursing students [2] and the NIC_CA‐Prof tool for Registered Nurses [27] evaluated competencies but did not incorporate direct patient feedback. While the NIC_CA Tool identified a predictive model of five composites—‘needs’, therapeutic relationship’, ‘problem‐solving’, ‘self‐confidence’ and ‘mental balance’—the NIC_CA‐Prof tool only identified four composites—‘basic nursing care’, ‘therapeutic relationship’, ‘problem management’ and ‘adaptation’. In this sense, nursing students, immersed in a learning environment, often have an idealised view of the caring interaction, influenced by theory and limited experiences in clinical practice [48]. Registered Nurses, on the other hand, with established experience in the field, face the daily reality of practice, where factors such as workload, stress and institutional expectations may influence their perception and performance in the caring interaction [49]. Patients, on the other hand, experience this interaction from a unique and personal perspective, based on their needs, vulnerabilities and expectations about the care they receive [50]. It is also important to note that competence in developing caring interaction evolves throughout a nurse's career. What a student nurse considers an optimal caring interaction may differ substantially from the view of an experienced nurse [51]. These differences in perspectives underscore the complexity of the caregiving interaction and suggest that there is a diversity of interpretations of what constitutes an effective and successful interaction [52]. Moreover, the care interaction is influenced by multiple contextual factors, such as institutional policies, organisational culture and the demographic and socio‐cultural characteristics of both patients and health professionals [53]. These variables can amplify differences in perceptions and expectations about care.

The findings of this study are also consistent with the Caring Life Course Theory (CLCT), which conceptualises care as a progressive, relational process shaped by evolving patient experiences and healthcare interactions [54] and aligns with its Care Biography approach, which integrates historical patient experiences into contemporary care planning [55]. These findings reinforce the importance of continuity and relational depth in patient‐centred care models.

Moreover, while existing patient experience metrics largely rely on general satisfaction surveys [8], the NIC_CA‐Patient tool provides a structured and validated tool that captures specific relational, problem‐solving, and adaptive dimensions of nurse–patient interactions. The increasing role of real‐time AI‐based predictive modelling in healthcare assessments, such as PROLIFERATE_AI, has further demonstrated the need for dynamic, patient‐centred and feedback tools [56].

Furthermore, the NIC_CA‐Patient predictive model provides a more nuanced alternative to traditional PREMs, which typically rely on overall satisfaction ratings or general impressions of care. By capturing the sequential and relational dimensions of nurse–patient interactions, the tool enables identification of specific phases where breakdowns in caring interaction may occur. This granularity offers added value in the continuous monitoring and improvement of care quality and supports its potential integration into national or institutional quality indicator frameworks alongside other PROMs and PREMs [57, 58].

### 4.2 Implications for Policy, Practice and Education

The results of this study offer important implications for healthcare policy, clinical practice and nursing education. Given that therapeutic relationship is the strongest predictor of problem management and adaptation, structured interventions to enhance nurse–patient communication and trust‐building should be prioritised. Healthcare institutions should integrate NIC_CA‐Patient as a standardised evaluation tool to assess and improve relational care quality across hospitals and clinical settings [56]. Current healthcare performance metrics often prioritise clinical efficiency over relational quality measures, despite strong evidence that nurse–patient interactions impact treatment adherence, patient satisfaction and emotional well‐being [15, 16, 59].

The adoption of AI‐driven predictive modelling, as presented in this work and emerging methods, could complement traditional patient feedback surveys by providing real‐time monitoring of patient experiences using sentiment analysis and machine learning algorithms [29, 56]. Integrating AI with structured tools like NIC_CA‐Patient would enable more proactive care interventions, allowing for dynamic adjustments to relational care strategies based on real‐time patient feedback.

From an educational perspective, our work reinforces the need for longitudinal, patient‐centred training in nursing curricula. Traditional communication training often focuses on early‐career nurses, but the progressive nature of caring interactions suggests that structured, multi‐stage training programmes are needed. Integrating patient experiential frameworks into nursing education could further enhance students' understanding of the evolving nature of patient care expectations [55].

In addition, this model offers educational value as a teaching tool by translating abstract caring concepts into measurable and observable components. It facilitates the assessment and development of caring competences in clinical education, consistent with contemporary pedagogical movements that integrate patient feedback and lived experience into the curricula [60, 61]. Its predictive nature enables educators to identify key phases in the development of the caring interaction, offering actionable guidance for both formative assessment and reflective practice.

While this study focused on patients admitted to internal medicine units, the theoretical structure and content of the NIC_CA‐Patient model suggest promising applicability to other clinical settings. Given the central role of relational care across diverse healthcare environments, further validation studies could explore the tool's adaptability and relevance in contexts where patients' needs, care trajectories, and communication dynamics may differ substantially [62].

### 4.3 Limitations

Despite the strong psychometric properties of the NIC_CA‐Patient model, some limitations should be acknowledged. First, although the EFA provided a theoretically coherent three‐factor structure for the NIC_CA‐Patient instrument, this study did not aim to confirm construct validity through CFA. Future studies should address this gap by performing confirmatory factor analysis using a new independent sample, as recommended in psychometric research [63]. Such validation would strengthen both the theoretical and empirical foundations of the instrument and provide further evidence of its dimensional stability.

Second, the study was conducted in eight hospitals within the Andalusian Health Care System, which may limit generalisability to other healthcare settings and cultural contexts. Future research should validate NIC_CA‐Patient across diverse healthcare systems to assess cross‐cultural reliability and applicability. Moreover, the cross‐sectional design of this study prevents an analysis of how caring interactions evolve. While the model establishes a strong predictive relationship between factors, longitudinal studies tracking patient experiences at multiple healthcare touchpoints are needed to determine how relational care perceptions change over extended periods.

Third, this study relied on self‐reported patient data, which introduces the possibility of recall bias and social desirability bias. While the NIC_CA‐Patient tool mitigates this through its validated structure, integrating AI‐based sentiment analysis [56] could enhance data reliability by capturing real‐time patient feedback. Additionally, the study did not explicitly analyse organisational‐level constraints such as nurse workload, staffing levels and institutional policies, which may impact nurse–patient interactions. Future research should explore how systemic healthcare factors influence relational care quality and whether policy interventions can mitigate negative organisational effects.

### 4.4 Future Research Directions

Future studies should focus on longitudinal validation of the NIC_CA‐Patient model across different healthcare settings to assess its stability over time. Implementing a multi‐centre, international study would provide cross‐cultural insights into patient perceptions of caring interactions. Research should also explore how AI‐driven patient experience tracking can enhance predictive modelling for relational care quality assessment. AI‐based approaches could offer continuous patient feedback, enabling adaptive, real‐time care interventions.

Further studies should investigate how institutional and policy factors influence caring interactions. Examining how workload distribution, nurse–patient ratios and staffing policies impact relational care would provide valuable insights into systemic constraints on caring interactions. Finally, research should assess how structured communication training at different career stages influences patient experiences. As caring competencies evolve over a nurse's career, evaluating the impact of longitudinal training interventions could inform nursing education reform and workforce development strategies.

## 5 Conclusion

This study presents a validated, patient‐centred model of caring interactions, offering a structured approach to measuring and enhancing nurse–patient relationships in clinical settings. The findings emphasise the central role of therapeutic relationship in facilitating problem management and adaptation, reinforcing the importance of relational engagement in nursing care. By integrating contemporary care frameworks and providing quantitative validation of patient perspectives, this study contributes to the advancement of caring sciences and offers valuable implications for clinical practice, healthcare policy and nursing education.

## Author Contributions

Regina Allande‐Cussó, María Alejandra Pinero‐De Plaza, Juan Gómez‐Salgado and Ana María Porcel‐Gálvez conceptualized and drafted the manuscript; Regina Allande‐Cussó, María Alejandra Pinero‐De Plaza, Juan Gómez‐Salgado and Ana María Porcel‐Gálvez conducted the data analysis; Regina Allande‐Cussó and Ana María Porcel‐Gálvez designed and critically reviewed the manuscript, which was also reviewed by María Alejandra Pinero‐De Plaza and Juan Gómez‐Salgado All of the authors read and approved the final version of the manuscript.

## Disclosure

Permission to Reproduce Material From Other Sources: Not applicable.

## Ethics Statement

Ethical approval was obtained from the Ethics Committee of the Andalusian government (approval code: 0840‐N‐22).

## Consent

Informed consent was obtained from all of the participants in this study.

## Conflicts of Interest

The authors declare no conflicts of interest.

## Supporting information


**Data S1:** Supporting Information (1)

**Data S2:** Supporting Information (2)

## Data Availability

The data that support the findings of this study are available from the corresponding author upon reasonable request.
