# Supplementary material for: Measuring the Science of Caring: A Patient‐Centred Predictive Model for the Caring Interaction
Source: Scand J Caring Sci. 2025 Sep 9;39(3):e70110. doi: 10.1111/scs.70110 (PMC12418724; doi:10.1111/scs.70110)
Supplement: Supplementary file 1 — Data S1: Supporting Information (1) [file SCS-39-0-s001.docx]

**SUPPLEMENTARY MATERIAL (1)**

This table compares the original CNPI items (Spanish validated version^1,2^) with their adapted versions for hospitalised patients in the NIC_CA-Patient questionnaire. The final column summarises the main linguistic and conceptual modifications.

| Original Item (CNPI Scale^1^) | Original Item (Validated Version^2^) | Adapted Item (Patient Version) | Main Change |
| --- | --- | --- | --- |
| CNPI 7 | Le ha demostrado que acudirá siempre que lo necesite | Mi enfermera/o me ha demostrado que acudirá siempre que lo necesite | Minor wording adaptation for patient-centred phrasing |
| CNPI 9 | Le anima a tener confianza en usted mismo/a | Mi enfermera/o me ha animado a tener confianza en mí mismo/a | Minor wording adaptation for patient-centred phrasing |
| CNPI 10 | Llama la atención hacia aspectos positivos sobre usted o su situación de salud | Mi enfermera/o ha resaltado aspectos positivos sobre mí o sobre mi situación de salud | Minor wording adaptation for patient-centred phrasing |
| CNP 11 | Le anima a tener esperanza | Mi enfermera/o me ha animado a tener esperanza | Minor wording adaptation for patient-centred phrasing |
| CNP 12 | Enfatiza sus esfuerzos | Mi enfermera/o ha valorado mis esfuerzos | Minor wording adaptation for patient-centred phrasing |
| CNPI 18 | Le ayuda a encontrar motivación para mejorar su situación de salud o sentirse mejor | Mi enfermera/o me ha ayudado a encontrar motivación para mejorar mi situación de salud o sentirme mejor | Minor wording adaptation for patient-centred phrasing |
| CNPI 19 | Le ha explicado cómo se sienten sus familiares respecto a su situación de salud | Mi enfermera/o me ha explicado cómo se sienten mis familiares respecto a mi situación de salud | Minor wording adaptation for patient-centred phrasing |
| CNPI 27 | Mantiene informados a sus allegados, si usted lo ha consentido | Mi enfermera/o ha mantenido informados a mis allegados, si yo lo he consentido | Minor wording adaptation for patient-centred phrasing |
| CNPI 33 | Le anima a hablar de sus pensamientos o sentimientos | Mi enfermera/o me ha animado a hablar de mis pensamientos o sentimientos | Minor wording adaptation for patient-centred phrasing |
| CNPI 34 | Le ha ayudado a establecer objetivos realistas respecto a su situación de salud | Mi enfermera/o me ha ayudado a establecer objetivos realistas respecto a mi situación de salud | Minor personalization towards the patient perspective |
| CNPI 35 | Le ayuda a afrontar el estrés o la angustia | Mi enfermera/o me ha ayudado a afrontar el estrés o la angustia | Minor wording adaptation for patient-centred phrasing |
| CNPI 36 | Le ayuda a ver las cosas desde otro punto de vista | Mi enfermera/o me ha ayudado a ver las cosas desde otro punto de vista | Minor wording adaptation for patient-centred phrasing |
| CNPI 39 | Le facilita encontrar solución a sus problemas | Mi enfermera/o me ha facilitado encontrar solución a mis problemas | Minor wording adaptation for patient-centred phrasing |
| CNPI 44 | Le permite y facilita hacer preguntas sobre su situación de salud | Mi enfermera/o me ha permitido y facilitado hacer preguntas sobre mi situación de salud | Minor wording adaptation for patient-centred phrasing |
| CNPI 45 | Le ha dado la oportunidad de practicar el autocuidado, si su situación lo permite | Mi enfermera/o me ha dado la oportunidad de practicar el autocuidado, si mi situación lo permite | Minor wording adaptation for patient-centred phrasing |
| CNPI 46 | Respeta su ritmo y tiempo, cuando está usted hablando | Mi enfermera/o ha respetado mi ritmo y tiempo, cuando yo estaba hablando | Minor wording adaptation for patient-centred phrasing |
| CNPI 47 | Le ha enseñado cómo programar la medicación o le ha explicado el tratamiento que le administraba | Mi enfermera/o me ha enseñado cómo programar la medicación o me ha explicado el tratamiento que me administraba | Minor wording adaptation for patient-centred phrasing |
| CNPI 51 | Le ha explicado los efectos adversos que usted puede sufrir, y cómo evitarlos, si cabe | Mi enfermera/o me ha explicado los efectos adversos que puedo sufrir, y cómo evitarlos, si cabe | Minor wording adaptation for patient-centred phrasing |
| CNPI 52 | Ha comprobado si el tratamiento le ha aliviado sus síntomas | Mi enfermera/o ha comprobado si el tratamiento me ha aliviado los síntomas | Minor wording adaptation for patient-centred phrasing |
| CNPI 55 | Ha respetado su intimidad | Mi enfermera/o ha respetado mi intimidad | Minor wording adaptation for patient-centred phrasing |
| CNPI 56 | Le ha ayudado con los cuidados que usted no podría realizar de forma autónoma | Mi enfermera/o me ha ayudado con los cuidados que no podría realizar de forma autónoma | Minor personalization towards patient perspective |
| CNPI 64 | La enfermera ha tenido en cuenta sus necesidades básicas (comer, beber, higiene, vestirse, movilidad), le ha ayudado con ellas y ha respetado sus deseos a cerca de las mismas | Mi enfermera/o ha tenido en cuenta mis necesidades básicas (comer, beber, higiene, vestirme, movilidad), me ha ayudado con ellas y ha respetado mis deseos sobre las mismas | Minor personalization towards patient perspective |
| CNPI 65 | Le ha ayudado a sentirse bien consigo mismo/a, cómodo/a y seguro/a | Mi enfermera/o me ha ayudado a sentirme bien conmigo mismo/a, cómodo/a y seguro/a | Minor personalization towards patient perspective |
| CNPI 66 | Le ha preguntado si quiere hablar con alguien, como un sacerdote u otros | Mi enfermera/o me ha preguntado si quería hablar con alguien, como un sacerdote u otros | Minor wording adaptation for patient-centred phrasing |
| CNPI 67 | Le ha ayudado a explorar lo que es importante en su vida | Mi enfermera/o me ha ayudado a explorar lo que es importante en mi vida | Minor personalization towards patient perspective |
| CNPI 68 | Le ha facilitado encontrar un significado o comprender su situación de salud | Mi enfermera/o me ha facilitado encontrar un significado o comprender mi situación de salud | Minor wording adaptation for patient-centred phrasing |
| CNPI 69 | Le ha ayudado a recuperar un cierto equilibrio emocional | Mi enfermera/o me ha ayudado a recuperar un cierto equilibrio emocional | Minor personalization towards patient perspective |
| CNPI 70 | Ha tenido en consideración sus necesidades espirituales | Mi enfermera/o ha tenido en consideración mis necesidades espirituales | Minor wording adaptation for patient-centred phrasing |

1.Allande R, Macías J, Porcel A. Transcultural adaptation into Spanish of the Caring Nurse-Patient Interactions for assessing nurse-patient relationship competence. Enferm Clin. 2020;30(1):42–6.

2.Allande-Cussó R, Gómez-Salgado J, Macías-Seda J, Porcel-Gálvez A. Assessment of the nurse-patient interaction competence in undergraduate nursing students. Nurse Educ Today. 2021;96(21):104627.
