# Supplementary material for: Measuring the Science of Caring: A Patient‐Centred Predictive Model for the Caring Interaction
Source: Scand J Caring Sci. 2025 Sep 9;39(3):e70110. doi: 10.1111/scs.70110 (PMC12418724; doi:10.1111/scs.70110)
Supplement: Supplementary file 2 — Data S2: Supporting Information (2) [file SCS-39-0-s002.docx]

**SUPPLEMENTARY MATERIAL (2)**

This table presents the final 18 items of the NIC_CA-Patient questionnaire, each linked to its corresponding original CNPI item number. It includes the English and Spanish versions adapted for hospitalised adults.

| CNPI Original Item | NIC_CA (English version) | NIC_CA (Spanish version) |
| --- | --- | --- |
| 7 | Show that he/she is there for you if you need her/him | Mi enfermera/o me ha demostrado que acudirá siempre que lo necesite |
| 9 | Encourage you to be hopeful, when it is appropriate. | Mi enfermera/o me ha animado a tener confianza en mí mismo/a |
| 10 | Draw your attention to positive aspects concerning you and your state of health. | Mi enfermera/o ha resaltado aspectos positivos sobre mí o sobre mi situación de salud |
| 11 | Encourage you to be hopeful, when it is appropriate. | Mi enfermera/o me ha animado a tener esperanza |
| 12 | Emphasize your efforts. | Mi enfermera/o ha valorado mis esfuerzos |
| 18 | Help you to find motivation to improve your state of health. | Mi enfermera/o me ha ayudado a encontrar motivación para mejorar mi situación de salud o sentirme mejor |
| 35 | Help you cope with stress or distress | Mi enfermera/o me ha ayudado a afrontar el estrés o la angustia |
| 36 | Help you to see things from a different point of view. | Mi enfermera/o me ha ayudado a ver las cosas desde otro punto de vista |
| 45 | Gave you the opportunity to practise self-care, if your condition allowed. | Mi enfermera/o me ha dado la oportunidad de practicar el autocuidado, si mi situación lo permite |
| 51 | Explained the possible side effects you could experience and how to avoid them, if applicable | Mi enfermera/o me ha explicado los efectos adversos que puedo sufrir, y cómo evitarlos, si cabe |
| 52 | Check if your medications soothe your symptoms (e.g., nausea, pain, constipation, anxiety, etc.). | Mi enfermera/o ha comprobado si el tratamiento me ha aliviado los síntomas |
| 55 | Respect your privacy | Mi enfermera/o ha respetado mi intimidad |
| 64 | Take your basic needs into account (e.g., sleeping, hygiene, etc.) | Mi enfermera/o ha tenido en cuenta mis necesidades básicas (comer, beber, higiene, vestirme, movilidad), me ha ayudado con ellas y ha respetado mis deseos sobre las mismas |
| 65 | Helps you to feel well in your condition. | Mi enfermera/o me ha ayudado a sentirme bien conmigo mismo/a, cómodo/a y seguro/a |
| 66 | He/she has asked you if you would like to talk to someone, such as a priest or others | Le ha preguntado si quiere hablar con alguien, como un sacerdote u otros |
| 68 | Help you to explore the meaning that you give to their health condition | Mi enfermera/o me ha facilitado encontrar un significado o comprender mi situación de salud |
| 69 | Help you to look for a certain equilibrium/balance in your lives. | Mi enfermera/o me ha ayudado a recuperar un cierto equilibrio emocional |
| 70 | Take into consideration your spiritual needs (e.g., prayer, meditation, participation in certain rites, etc.). | Mi enfermera/o ha tenido en consideración mis necesidades espirituales |
